# Construction and Validation of a Novel Eight-Gene Risk Signature to Predict the Progression and Prognosis of Bladder Cancer

**DOI:** 10.3389/fonc.2021.632459

**Published:** 2021-06-29

**Authors:** Ruiliang Wang, Zongtai Zheng, Shiyu Mao, Wentao Zhang, Ji Liu, Cheng Li, Shenghua Liu, Xudong Yao

**Affiliations:** Department of Urology, Shanghai Tenth People′s Hospital, Tongji University School of Medicine, Shanghai, China

**Keywords:** bladder cancer, LASSO, WGCNA, nomogram, malignant progression, prognosis, risk signature

## Abstract

The progression from non-muscle-invasive bladder cancer (NMIBC) to muscle-invasive bladder cancer (MIBC) increases the risk of death. It is therefore important to find new relevant molecular models that will allow for effective prediction of the progression and prognosis of bladder cancer (BC). Using RNA-Sequence data of 49 BC patients in Shanghai tenth people’s hospital (STPH) and weighted gene co-expression network analysis methods, a co-expression network of genes was developed and three key modules associated with malignant progression were selected. Based on the genes in three key modules, an eight-gene risk signature was established using univariate Cox regression and the Least absolute shrinkage and selection operator Cox model in The Cancer Genome Atlas Program (TCGA) and validated in validation sets. Subsequently, a nomogram based on the risk signature was constructed for prognostic prediction. The mRNA and protein expression levels of eight genes in cell lines and tissues were further investigated. The novel eight-gene risk signature was closely related to the malignant clinical features of BC and could predict the prognosis of patients in the training dataset (TCGA) and four validation sets (GSE32894, GSE13507, IMvigor210 trial, and STPH). The nomogram showed good prognostic prediction and calibration. The mRNA and protein expression levels of the eight genes were differentially expressed in cell lines and tissues. In our study, we established a novel eight-gene risk signature that could predict the progression and prognoses of BC patients.

## Introduction

Globally, bladder cancer (BC) is ranked 4^th^ as the most commonly diagnosed cancer in men (1). Among BC patients, 75% are diagnosed with non-muscle invasive bladder cancer (NMIBC) whereas 25% are diagnosed with muscle-invasive bladder cancer (MIBC). However, >30% of patients with NMIBC develop recurrence and progresses to MIBC within 5 years after diagnosis (2). Moreover, three-quarters of MIBC patients develop distant metastases with their long-term survival rate being 15% (3).

For many decades, the gold standard for monitoring BC recurrence and progression is lifelong cystoscopy and cytology (4). However, cystoscopy is costly, invasive, and uncomfortable. In addition, urine cytology is of limited value due to low sensitivity, especially for low-grade lesions (5). Hence, many clinical index models and non-invasive marker tests to predict the recurrence and progression of BC have been investigated extensively in recent years. But the accuracy of most published models behaves poorly when used outside of the scope for which they were created (6–8). Furthermore, some previous risk signatures were constructed based on the gene microarray that contains limited expression levels of genes, which may limit the performance of the risk signatures. For these reasons, it is important to identify valuable models or biomarkers for evaluating the prognosis and monitor the progression of BC.

In this study, a new prognostic risk model based on eight differently expressed genes between MIBC and NMIBC is established for predicting the prognosis and progression of BC. The performance of the risk model was verified using gene expression data from the Gene Expression Omnibus (GEO), IMvigor210 trial, and our center. A prognostic nomogram based on the risk signature was built to predict the prognosis of BC. Moreover, real-time quantitative (RT-qPCR) and immunohistochemistry (IHC) were conducted to investigate the expression levels of eight genes in cell lines and BC samples.

## Materials and Methods

### Clinical Samples and RNA-Sequencing

A total of 49 BC patients (35 NMIBC and 14 MIBC) in Shanghai Tenth People’s Hospital (STPH) who underwent either transurethral resection of BC or radical cystectomy between November 2019 and April 2020 were included in this study. The following criteria were used to enroll patients in the training set were: (1) histologically confirmed BC; (2) availability of freshly collected tissue from surgery; and (3) availability of clinical data and prognostic information. Informed consent was obtained and ethical approval was granted by the Ethics Committee of Shanghai Tenth People’s Hospital. The workflow of RNA-Seq was performed as described in an earlier article, and the mRNA (RNA-sequence) Fragments Per Kilobase of transcript per Million Fragments (FPKM) standardized expression data was used for further analyses (9). Characteristic of patients with bladder cancer in STPH is shown in **Supplement Table 1**.

### Public Data Processing

Gene expression data with sufficient prognostic information from GSE32894 (n = 224) and GSE13507 (n = 165) datasets were downloaded from GEO (http://www.ncbi.nlm.nih.gov/geo) as a series of matrix file format that was earlier processed by the original authors using the MAS 5.0 algorithm. IMvigor210 (n = 298) with immunotherapy data and clinical information were obtained from the IMvigor210CoreBiologies R package. The latest TCGA data containing mRNA FPKM standardized expression data, clinical features, and follow-up information was downloaded using GDC API. Characteristic of patients with BC in TCGA is shown in **Supplement Table 1**. The representative immunohistochemistry (IHC) of genes in both normal human bladder tissues and tumor tissues were obtained from the Human Protein Atlas (HPA) (http://www.proteinatlas.org/).

### Establishment of Prediction Models and Bioinformatics Analyses

#### Weighted Gene Co−Expression Network Analysis

The “limma” R package v3.46 was adopted to screen for differentially expressed genes (DEGs, P value <0.05 and |log2FC| ≥ 0.5) between 35 NMIBC and 14 MIBC samples in STPH. A weighted gene co-expression network analysis (WGCNA) was constructed (10) using the “WGCNA” R package v1.68 based on the DEGs. WGCNA was performed as follows: (1) Outlier samples were omitted to increase the reliability of the weighted gene co-expression network, (2) An appropriate β value was selected using 0.85 as the degree of independence (R^2^), (3) A weighted adjacent matrix was transformed into a topological overlap matrix (TOM) to determine the network connectivity of the genes, (4) Genes with similar expression profiles were classified into gene modules based on the average linkage hierarchical clustering following the TOM-based dissimilarity measure, (5) All genes were represented by the expression of module eigengenes (MEs) in a given module. Modules that were highly correlated with NMIBC/MIBC subtype (|r| ≥ 0.3) were selected for further analyses, and (6) the genes in the selected modules (brown, turquoise, and yellow) were extracted.

#### Construction of the Risk Signature

Based on hub genes extracted from WGCNA, univariate Cox regression analysis was conducted to select genes associated with overall survival (OS) in the TCGA dataset (11). Thereafter, the genes were subjected to Least absolute shrinkage and selection operator (LASSO) analysis with 10-fold cross-validation to construct a novel eight-gene risk signature using “glmnet” R package v2.0. Genes with minimal influence on the patient’s OS were removed whereas those with non-zero coefficients were selected. The risk signature for each patient was calculated as follows: risk signature = Coef_1_ × expression of gene_1_ + Coef _2_ × expression of gene_2_ + … + Coef_m_ × expression of gene m. Coef denotes the corresponding coefficient of the gene. The “survminer” R package v4.6 was used to evaluate the optimal cutoff value of the risk signature in each cohort. The established optimal cutoff value was used to divide patients into low- and high-risk groups accordingly.

#### Gene Sets Enrichment Analysis

GSEA (http://www.broadinstitute.org/gsea/index.jsp) analysis by using TCGA data was conducted to compare pathways between patients in high- and low-risk groups. Signaling pathways with P < 0.05 and a false discovery rate <0.25 were regarded as statistically significant.

#### Gene Set Cancer Analysis

Gene Set Cancer Analysis (GSCALite, http://bioinfo.life.hust.edu.cn/web/GSCALite/) provides a single nucleotide variation (SNV) module through the maftools (12). Herein, we employed the SNV module to analyze and visualize the SNV of the eight genes in BC.

### Eight-Gene Risk Signature Validation

To evaluate the generalizability of the model, four validation datasets (GSE32894, GSE13507, IMvigor210, and STPH) were analyzed using the same formula and coefficients. The Kaplan-Meier curves of the validation datasets were separately drawn before analyzing the prognostic performance of the novel eight-gene risk signature. Furthermore, the violin plot was used to verify the relationship between the risk signature and NMIBC/MIBC subtypes.

### Development of the Nomogram

A nomogram was developed from the factors that were significant in the final multivariate Cox regression analyses. The ROC, concordance index (C-index), and calibration plots were conducted to assess the performance of the nomogram. The nomogram, C-index, and calibration plots were generated using the “Rms” R package v6.1. Furthermore, a decision curve analysis (DCA) was employed to ascertain the net benefits of nomogram and other crucial prognostic factors.

### Cell Culture

The human normal bladder epithelial cell line SV-HUC-1 and BC cell line (13, 14) (two grade 1 cancer cell line SW780/RT4, a grade 2 cancer cell line 5637, and a grade 3 cancer cell line T24) obtained from Shanghai Cell Bank of the Chinese Academy of Sciences (Shanghai, China) were used for *in vitro* experiments. The normal bladder epithelial cell line of SV-HUC-1 was cultured in F12K (Gibco; Thermo Fisher Scientific, Inc.). BC cell lines of 5637 and T24 were cultured in RPMI-1640 (Gibco; Thermo Fisher Scientific, Inc.). RT4 was cultured in McCoy’s 5A (Gibco; Thermo Fisher Scientific, Inc.). SW780 was cultured in Leibovitz’s medium (Gibco; Thermo Fisher Scientific, Inc.). Each media was supplemented with 10% FBS (Gibco; Thermo Fisher Scientific, Inc.) and 1% P/S (Gibco; Thermo Fisher Scientific, Inc.). Cell culture reagents were bought from Gibco and the cells were cultured at 37˚C and 5% CO_2_.

### RT-qPCR

TRIzol reagent (Invitrogen, Thermo Scientific, Shanghai, China) was used for extracting RNA from the cell lines. The cDNA Synthesis SuperMix Kit (Cat No.11141ES60; Yeasen, Shanghai. China) was used to synthesize cDNA which was subjected to qPCR using qPCR SYBR Green Master Mix KIT (Cat No. 11203ES03; Yeasen, Shanghai. China). The gene expression level was normalized to the expression of GAPDH mRNA. Primer sequences used are presented in **Supplement Table 2**.

### Statistical Analysis

The Wilcoxon rank-sum test was used to identify the differential expressed genes between NMIBC and MIBC. The Student’s t-test or one-way ANOVA was used to evaluate the association of the risk signature with clinical characteristics. Kaplan-Meier survival analysis was performed, Cox regression models were built to estimate hazard ratios (HRs) between the high- and low-risk groups. The prediction performance of the risk signature in NMIBC/MIBC subtypes was determined from ROC curves. A heatmap and correlation matrix were generated using “Pheatmap” R package v1.0.12 and “corrplot” R package v0.84, respectively. SPSS 22.0 (SPSS, Armonk, NY, USA), R v3.6.1 (https://www.r-project.org/), and Graphpad Prism V7 (GraphPad Software, Inc.) were used for data analysis. A two-sided P value <0.05 was considered significant. The genes expression of cell lines were subjected to one-way ANOVA and then analyzed by Tukey’s pairwise comparison to identify expression differences.

## Results

### Data Acquisition and DEGs Selection

**Figure 1:**
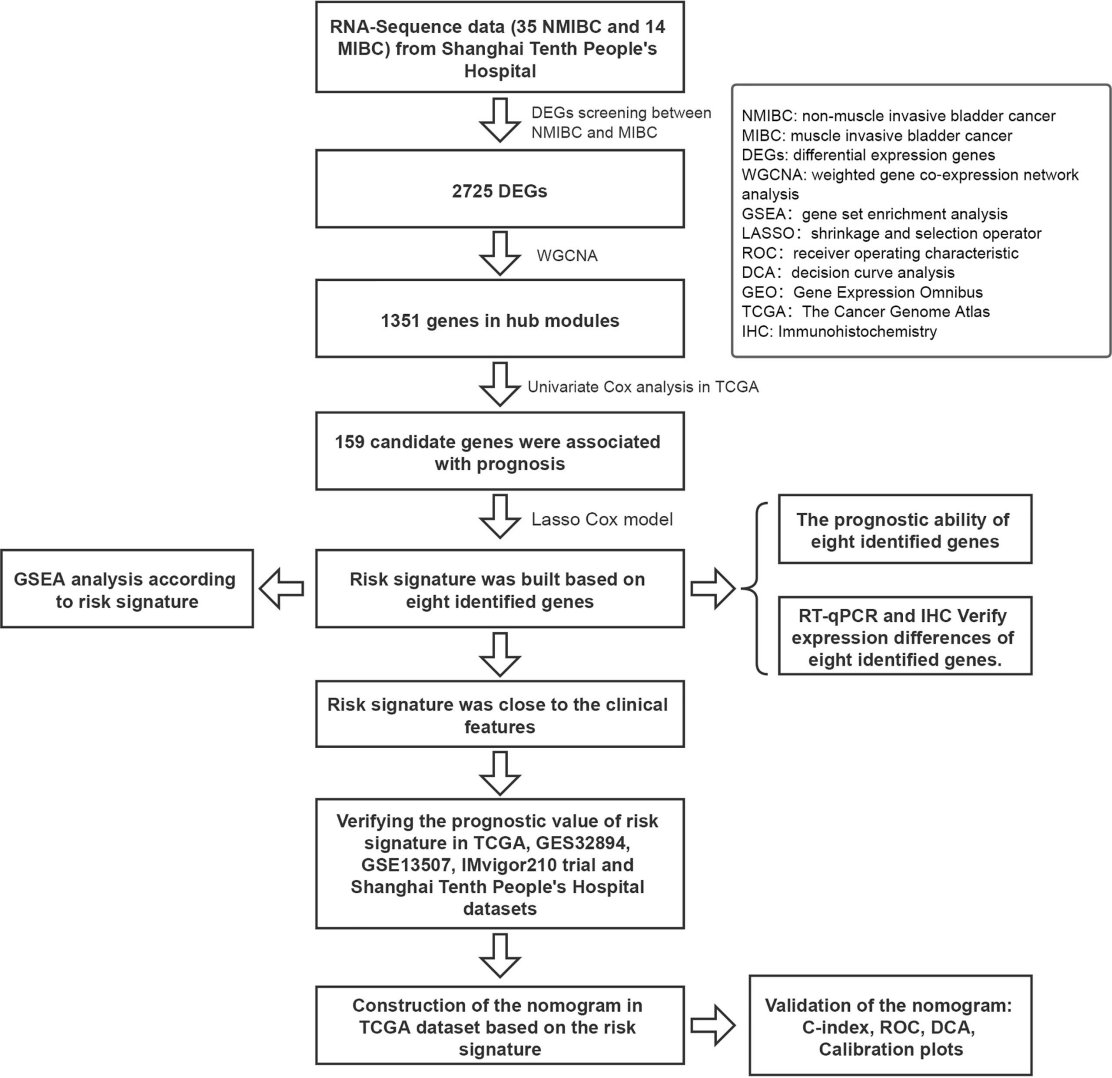
Flowchart showing the construction process of the risk signature model.

A flow diagram of the study is shown in **Figure 1**. A total of 49 BC patients (35 NMIBC and 14 MIBC) from STPH were enrolled for the study. From our findings, 2,725 DEGs were identified between NMIBC and MIBC.

### WGCNA Analysis Based on DEGs Between NMIBC and MIBC

**Figure 2:**
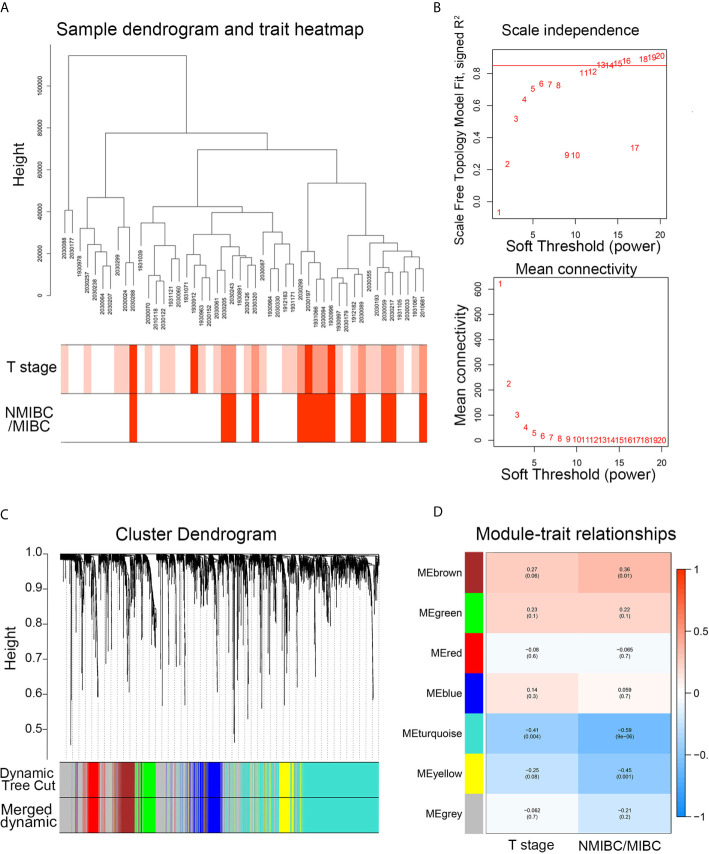
The WGCNA network construction and key module identification. **(A)** Sample dendrogram and trait indicator. The clustering was a visual result of calculations based on Pearson correlation coefficients between samples. The color intensity was proportional to T stage and NMIBC/MIBC subgroup of BC. **(B)** The analysis of topology for soft threshold powers and β = 13 (scale-free R^2^ = 0.85) was set as the soft thresholding for further adjacency calculation. **(C)** The original modules and merged modules are displayed at top and bottom of the clustering dendrogram. **(D)** Module-trait relationships between identified modules and clinical features. The numbers represent Pearson’s correlation between the clinical traits and modules. The numbers in the parentheses correspond to the p-value. WGCNA, weighted gene correlation network analysis; NMIBC, non-muscle-invasive bladder cancer; MIBC, muscle-invasive bladder cancer; ME, module.

After the hierarchical clustering analysis, one sample was removed as an outlier. A co-expression network was constructed using 48 BC samples with complete clinical T stage and NMIBC/MIBC subgroups (**Figure 2A**). With the chosen power of β = 13 (scale-free R^2^ = 0.85) as the soft-thresholding (**Figure 2B**), seven modules were obtained (**Figure 2C**). The highest module-trait association was found between three modules and the NMIBC/MIBC subgroups (brown, turquoise, and yellow) (**Figure 2D**). Subsequently, 1,351 genes from three key modules were selected for further analysis.

### Construction and Verification of the Risk Signature

Among the 1,315 genes, 159 were statistically significant (P values <0.05) in the univariate Cox analysis. In this study, genes with P values <0.05 and genes with P values <0.05/159 in univariate Cox analysis were selected for LASSO Cox regression analysis, respectively. The Lambda.1se penalty parameter was selected based on the 10-fold leave-one-out cross-validation in accordance with the minimum criteria. In this way, two risk signatures were developed. As for risk signature built based on genes with P values <0.05/159 in univariate Cox analysis, five genes (*CD96*, *IP6K2*, *TRIM38*, *DDX39B*, and *DDB1*) with non-zero coefficients were obtained by LASSO Cox regression analysis (**Supplement Figures 1A–C**). According to the aforementioned formula, risk signatures for each patient were calculated. Kaplan-Meier analysis showed that BC patients with high-risk scores had significantly poorer OS and disease-free survival (DFS) than BC patients in the low-risk group (P < 0.001) in TCGA (**Supplement Figures 1D, E**). We further investigated the prognostic value of the five-gene risk signature in four validation sets. The risk signature still had the prognostic ability in GSE32894 (P = 0.036), but not in GSE13507 (P = 0.878), IMvigor210 (P = 0.661), and STPH (P = 0.59) (**Supplement Figures 1F–I**).

**Figure 3:**
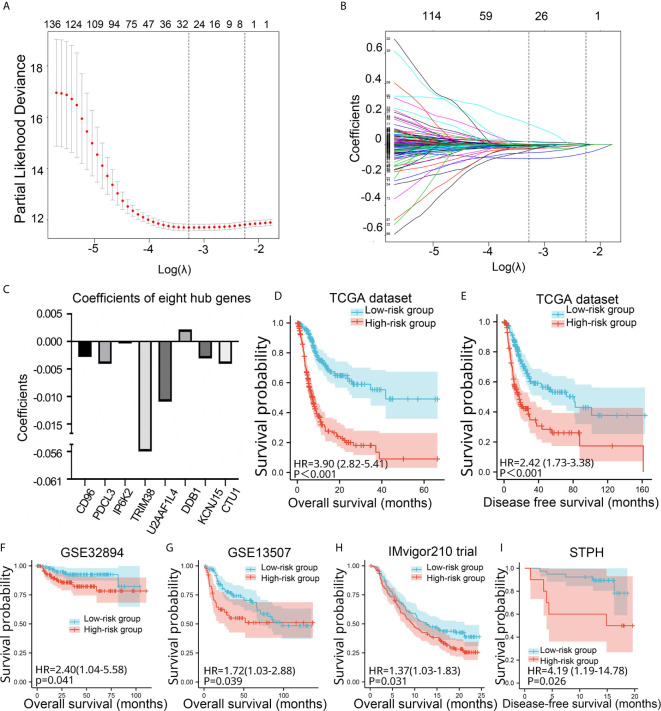
Construction and validation of the eight-gene risk signature. **(A)** Plots of the cross-validation error rates. **(B)** Distribution of LASSO coefficients of OS-associated genes. **(C)** Coefficient values of the eight genes. **(D–I)** Kaplan-Meier curves for BC patients assigned to high- and low-risk groups in TCGA, GSE32894, GSE13507, IMvigor210 trial, and STPH. LASSO, least absolute shrinkage and selection operator; TCGA, The Cancer Genome Atlas; BC, bladder cancer; NMIBC, non-muscle-invasive bladder cancer; MIBC, muscle-invasive bladder cancer; OS, overall survival; DFS, disease-free survival; STPH, Shanghai Tenth People’s Hospital.

As for risk signature built based on genes with P values <0.05 in univariate Cox analysis, eight genes (*CD96*, *PDCL3*, *IP6K2*, *TRIM38*, *U2AF1L4*, *DDB1*, *KCNJ15*, and *CTU1*) with non-zero coefficients were obtained (**Figures 3A–C**). Kaplan-Meier analysis revealed that BC patients in the high-risk group showed significantly poorer OS and DFS than BC patients in the low-risk group (P < 0.001) in TCGA (**Figures 3D, E**). We further validated the prognostic value of the risk signature in four validation sets. The risk signature still had the prognostic ability in GSE32894 (P = 0.041), GSE13507 (P = 0.039), and IMvigor210 (P = 0.031) (**Figures 3F–I**). In addition, patients in the high-risk group showed significantly poorer DFS than those in the low-risk group in STPH (P = 0.026, **Figure 3H**). Because the eight-gene risk signature had novel prognostic performance in validation data sets, it was selected for further analyses. The prognostic performance of eight genes was assessed individually. Results showed that their prognostic values were statistically significant (**Supplement Figure 2A**).

Among the 411 BC samples with complete sequencing data in TCGA, mutations of the eight genes were only found in 29 independent samples in TCGA dataset. Notably, *CD96* and *DDB1* recorded the highest rates of mutations (28%) (**Supplement Figure 2B**). GSEA analysis showed that “Focal adhersion,” “pathway in cancer,” “WNT signal,” “bladder tumor,” “ECM receptor,” and “adherens junctions pathway” were enriched in the high-risk group (**Supplement Figure 3**).

### Association of the Novel Eight-Gene Risk Signature With Clinical Features

**Figure 4:**
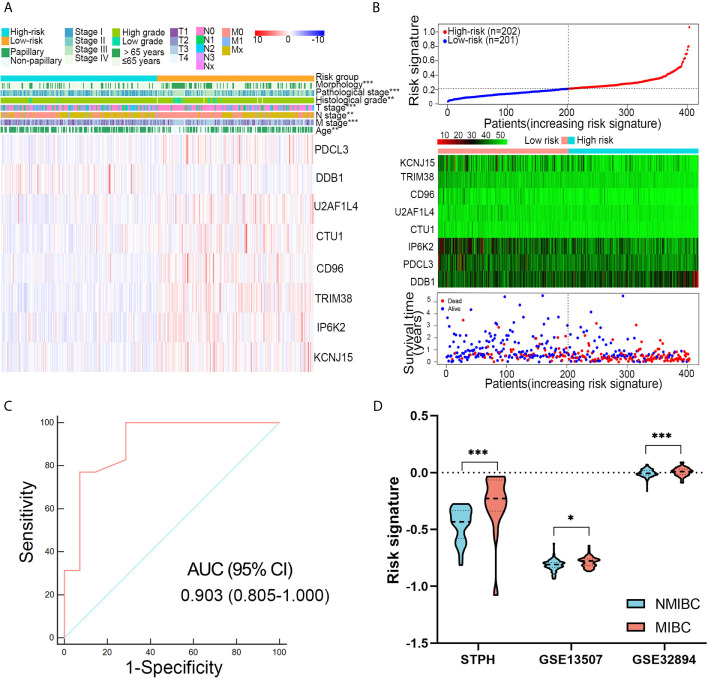
Associations between risk signature and clinical variables. **(A)** Clinical variables and expression levels of eight genes were compared between the low- and high-risk groups **(B)** Risk signatures distribution, OS status of each patient, and heatmaps of eight genes in TCGA dataset. **(C)** Receiver operating characteristic curve showed the discrimination power of the risk signature for NMIBC and MIBC in STPH dataset. **(D)** The distribution of risk signatures was compared between NMIBC and MIBC in STPH, GSE13507, and GSE32894 datasets. ^*^P < 0.05, ^**^P < 0.01, ^***^P < 0.001.

Patients with high-risk signatures showed the positive association with clinicopathological features, including age (P < 0.001), pathological stage (P < 0.001), histological grade (P < 0.01), T stage (P < 0.001), M stage (P < 0.01), N stage (P < 0.001), and morphology (P < 0.001) in TCGA dataset (**Figure 4A**). A dot plot based on the survival status of TCGA set indicated that patients in high-risk group had a higher mortality rate than those in low-risk group (**Figure 4B**). The ROC revealed that the risk signature could effectively differentiate NMIBC from MIBC with an AUC of 0.903 in STPH (**Figure 4C**). The distribution of risk signatures in training and validation sets was presented in detail in **Figure 4D**. It can be observed that MIBC patients have higher risk signatures than NMIBC patients (all P < 0.05).

### Construction and Calibration of the Nomogram

**Figure 5:**
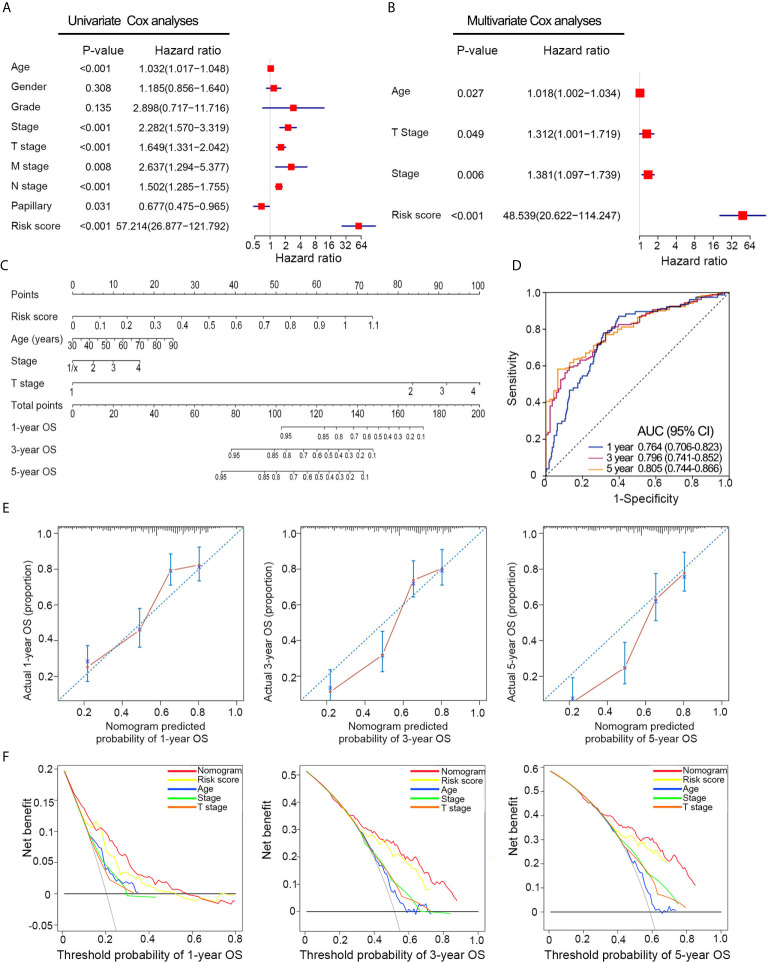
Nomogram construction based on the risk signature in TCGA dataset. **(A, B)** Univariate and multivariate Cox analyses indicated that the risk signature was significantly associated with OS. **(C)** Nomogram for predicting the probability of 1-, 3-, and 5-year OS. **(D)** ROC curve showed the predictive performance of 1-, 3-, and 5-year OS. **(E)** Calibration plots of the nomogram for predicting the probability of 1-, 3-, and 5-year OS. **(F)** DCA analysis showing the performance of the nomogram to predict the 1- 3-, and 5-year OS. OS, overall survival; ROC, Receiver operating characteristic; DCA, decision curves analyses; HR, hazard ratio; CI, confidence interval.

Univariate Cox analyses revealed that T stage, histological grade, age, and risk signature were significantly related to OS in the TCGA dataset (**Figure 5A**). After multivariate Cox analysis, risk signature, age, T stage, and pathological stage retained their prognostic value (**Figure 5B**). A nomogram was constructed for predicting the 1-, 3-, and 5-year OS based on the significant factors in multivariate Cox analyses (**Figure 5C**). The AUC values of ROC curves were 0.764 (1-year ROC), 0.796 (3-year ROC), and 0.805 (5-year ROC), whereas the C-index of the nomogram was 0.75 ± 0.02 (mean ± SD) for OS (**Figure 5D**). Besides, calibration plots proved that the prediction results of the nomogram were largely in agreement with the actual 1-, 3-, and 5-year OS (**Figure 5E**). Furthermore, compared with clinical features, the nomogram achieved the best clinical net benefit *via* DCA plot (**Figure 5F**).

### Validation of the Novel Eight-Gene Signature Through *In Vitro* Experiments and Public Database

**Figure 6:**
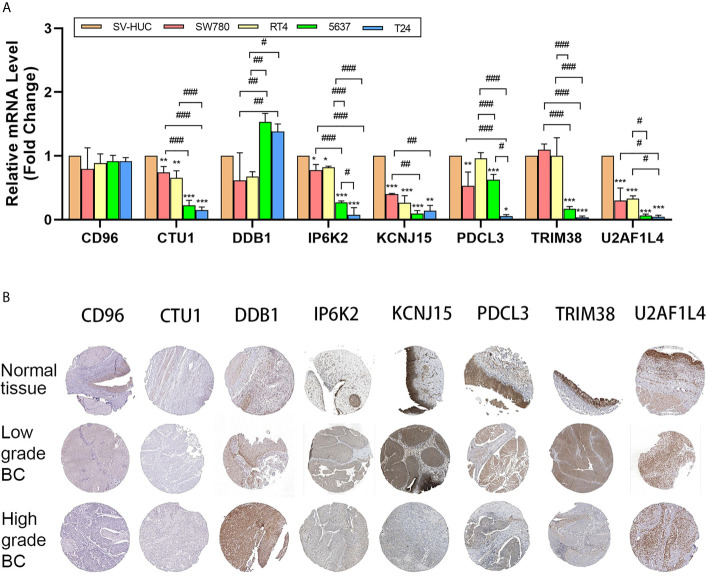
mRNA and protein level validation of eight genes. **(A)** RT-qPCR validates expression of eight genes in bladder cancer cell lines SW780, RT4, 5637, and T24 and normal bladder epithelial cell line SV-HUC-1. **(B)** IHC intensity of eight genes in normal bladder tissue and high/low-grade bladder cancer tissue from HPA. IHC, immunohistochemistry; BC, bladder cancer; *P < 0.05, **P < 0.01, ***P < 0.001, significant when compared to SV-HUC-1; ^#^P < 0.05, ^##^P < 0.01, ^###^P < 0.001, significant when pairwise comparisons among BC cell lines.

The mRNA expression pattern of the eight genes in five human BC cell lines (two grade 1 cancer cell line SW780/RT4, a grade 2 cancer cell line 5637, and a grade 3 cancer cell line T24) and one human normal bladder epithelial cell line (SV-HUC-1) was determined using RT-qPCR. Results showed that five of seven genes (*CTU1*, *DDB1*, *IP6K2*, *KCNJ15*, *PDCL3*, *TRIM38*, and *U2AF1L4*) were significantly different in transcriptional levels between BC and normal bladder cells (**Figure 6A**). Additionally, we noticed that the expression level of *CTU1*, *IP6K2*, *PDCL3*, *TRIM38*, and *U2AF1L4* was gradually decreased following the increases in the degree of malignancy. The representative IHC images of normal/tumor and low/high grade tissues from HPA revealed that the degree of staining intensity of *CD96*, *DDB1*, *IP6K2*, *PDCL3*, *TRIM38*, and *KCNJ15* were in correspondence with our predictions of coefficient. *U2AF1L4* showed medium to high IHC staining intensity in normal and tumor tissues. Neither normal tissues nor tumor tissues expressed the CUT1 protein (**Figure 6B**).

## Discussion

The BC progression occurs in approximately 15% of patients, and once patients develop progression, their prognoses are generally unfavorable. Thus, an accurate prediction of progression and prognoses are critically important in the management of low-grade BC or NMIBC (15). So far, several prediction models have been developed for BC (16, 17). Kim et al. used 1,320 genes to investigate the progression genes and four key genes were found. Catto et al. explored 11 progression-associated genes by using artificial intelligence with immunohistochemistry validation (18, 19). However, these models are based on their clinical centers without experimental validation or external validation, which limits the generalizability and reproducibility of the models. Based on the RNA-sequence data from our center and TCGA, we constructed an eight-gene risk signature using WGCNA and LASSO regression analyses. The results showed that the eight-gene risk signature had novel performance in prognostic stratification in training (TCGA) and validation (GSE32894, GSE13507, IMvigor210, and STPH) datasets, in which BC patients in the high-risk group showed significantly poorer prognoses than BC patients in the low-risk group. Taken together, the risk signature was considered as a useful prognostic tool for prognostic prediction in BC.

Then based on high/low risk of the model, GSEA analysis was performed. The results confirmed that “WNT signal” and “bladder tumor” pathways were highly enriched in high-risk group, suggesting the important role of the risk signature in tumorigenesis and malignant progression. GSCA results further indicated that these genes rarely mutated, implying that potential dysfunction in these genes may not be as a result of genetic mutations, but rather due to aberrant alterations at the transcriptional level (20). As for genes in the risk signature, some genes have been reported to play important roles in tumors in previous studies. For example, high expression of *CD96* in BC tissue, which was discovered in 1992, indicates high immune cells infiltration and hence killing of cancer cells (21, 22). Damage-specific DNA binding protein 1 (DDB1), as the only oncogene in our model, is a multifunctional protein. Previous studies showed that DDB1 interacts with several chaperones that regulate DNA repair mechanisms, cell cycle and gene transcription (23). DDB1 can directly interact with Cullin4B (CUL4B) to form the CUL4B-DDB1 complex which promotes ubiquitination and degradation of a variety of tumor suppressor factors hence enhance progression of osteosarcoma (24). The tripartite motif protein 38 (TRIM38), a member of the TRIM family, was found to confer protection against various cellular processes such as cell differentiation, proliferation, apoptosis, and antiviral defense. On the other hand, TRIM38 causes abnormal activation of NF-κB pathway thereby inhibiting cancer (25, 26).

We then integrated the risk signature and other independent prognostic factors to establish a nomogram for clinical practice. A nomogram that comprised the age, T stage, pathological stage, and risk signature was drawn to predict the OS. ROC showed that the nomogram could accurately predict the 1-, 3-, and 5-year OS of BC. Moreover, calibration plots and C-index confirmed the prognostic significance and predictive superiority of the nomogram. DCA revealed that compared with clinical features, the nomogram had the optimal enhanced clinical net benefit with wider threshold probabilities. All of these findings indicated that the risk signature-based nomogram may be a reliable evaluation tool to perform mortality risk identification in BC patients.

The followed is the experimental verification of the eight genes. The results of IHC assay in HPA and RT-qPCR revealed that most hub genes played important roles in tumorigenesis and progression. Nevertheless, the mRNA expression level (RT-qPCR in cell lines) and protein intensity (IHC in tissues) may not match completely due to the following reasons: (a) the tumor microenvironment between tumor tissues and cell lines was different. The occurrence and progression of tumors are a result of complex interactions (27). (b) The regulation of mRNA and protein expression is complex, and there is no clear relationship between mRNA and protein expression levels. (c) The construction of our signature was based on the NMIBC/MIBC, but we compare the low/high grade in the experiment part. As we all know, some NMIBC also belongs to the high-grade tumor.

Some limitations exist in the present study. First, the sample size from our center was small, and more samples with detailed clinicopathological and prognostic information are necessary to further investigate the performance of the risk signature in the prediction of the progression and prognosis of BC. Second, due to the relatively short follow-up time, we used the DFS to validate the prognostic value of the risk signature in STPH. Longer follow-up time is needed to further compare the OS of BC patients between the high- and low-risk groups. In addition, the functions of the eight genes in the malignant progression of BC have not been investigated. Further researches are needed to explore the mechanisms of the eight genes in tumor progression.

## Conclusion

This study demonstrated that a novel eight-gene risk signature could reliably predict the malignant progression and prognosis of BC patients. The constructed nomogram based on the eight-gene risk signature could accurately predict the survival of BC patients. Dysregulated expression of the eight genes was validated in BC samples and cell lines by IHC and RT-qPCR, respectively. Further functional studies and mechanistic experiments should be conducted to better understand the clinical value of the eight hub genes in BC.

## Data Availability Statement

Public data that support the findings of this study are openly available in TCGA (http://cancergenome.nih.gov/), NCBI GEO dataset GSE32894 (https://www.ncbi.nlm.nih.gov/geo/query/acc.cgi?acc=GSE32894) and GSE13507 (https://www.ncbi.nlm.nih.gov/geo/query/acc.cgi?acc=GSE13507). IMvigor210 with immunotherapy data and clinical information were obtained from the IMvigor210CoreBiologies R package. The processed data required to reproduce these findings cannot be shared at this time as the data also form part of an ongoing study. Requests to access the datasets should be directed to yaoxudong1967@163.com.

## Ethics Statement

The studies involving human participants were reviewed and approved by the Ethics Committee of Shanghai Tenth People’s Hospital. The patients/participants provided their written informed consent to participate in this study. Written informed consent was obtained from the individual(s) for the publication of any potentially identifiable images or data included in this article.

## Author Contributions

RW and ZZ conceived and designed the study. XY and SL acquired the funding. RW, SM, and WZ collected and collated the data. All authors analyzed and interpreted the data. RW wrote the manuscript, ZZ, SM, and WZ critically reviewed and revised the manuscript. JL, CL, and ZZ designed the tables and figures. All authors contributed to the article and approved the submitted version.

## Funding

This study was funded by the Shanghai Science Committee Foundation (grant number 19411967700) and the Shanghai Youth Science and Technology Talents Sailing Program (20YF1437200).

## Conflict of Interest

The authors declare that the research was conducted in the absence of any commercial or financial relationships that could be construed as a potential conflict of interest.

## Supplementary Material

Supplementary Figure 1 Construction and validation of the five-gene risk signature. **(A)** Plots of the cross-validation error rates. **(B)** Distribution of LASSO coefficients of OS-associated genes. **(C)** Coefficient values of the five genes. **(D–I)** Kaplan-Meier curves for BC patients assigned to high- and low-risk groups in TCGA, GSE32894, GSE13507, IMvigor210 trial, and STPH. LASSO, least absolute shrinkage and selection operator; TCGA, The Cancer Genome Atlas; BC, bladder cancer; NMIBC, non-muscle-invasive bladder cancer; MIBC, muscle-invasive bladder cancer; OS, overall survival; DFS, disease-free survival; STPH, Shanghai Tenth People’s Hospital.

Supplementary Figure 2 The prognostic ability and single nucleotide variation of eight genes using TCGA datasets. **(A)** Kaplan-Meier curves for overall survival of eight genes individually. **(B)** Single nucleotide variation distribution among eight genes. BC, bladder cancer.

Supplementary Figure 3 Gene Set Enrichment Analysis between patients in high- and low-risk groups. BC, bladder cancer.
