# Supplementary figures and images for: Construction and Validation of a Novel Eight-Gene Risk Signature to Predict the Progression and Prognosis of Bladder Cancer
Source: Front Oncol. 2021 Jun 29;11:632459. doi: 10.3389/fonc.2021.632459 (PMC8276675; doi:10.3389/fonc.2021.632459)

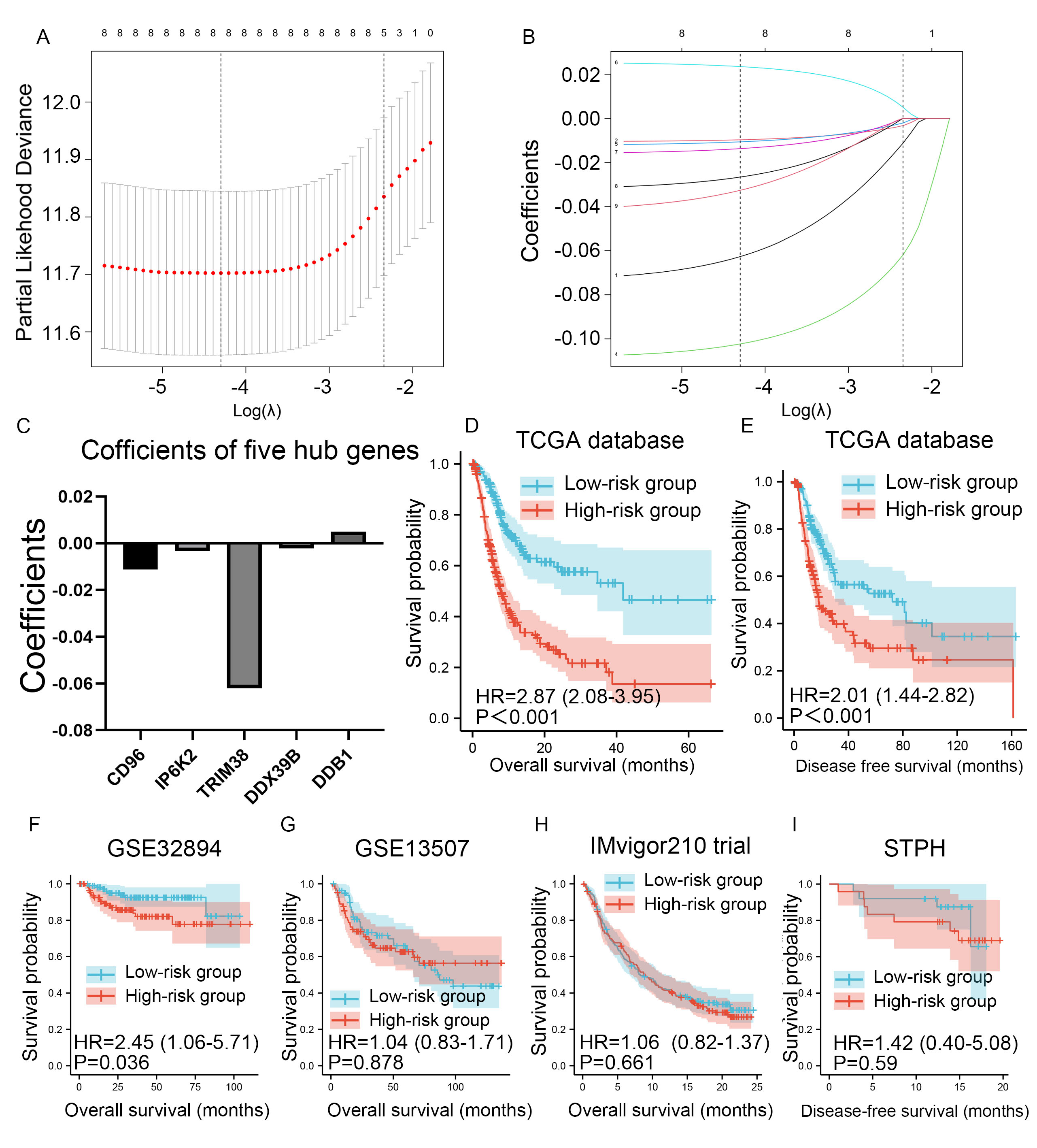

Supplement: Supplementary Figure 1 — Construction and validation of the five-gene risk signature. (A) Plots of the cross-validation error rates. (B) Distribution of LASSO coefficients of OS-associated genes. (C) Coefficient values of the five genes. (D–I) Kaplan-Meier curves for BC patients assigned to high- and low-risk groups in TCGA, GSE32894, GSE13507, IMvigor210 trial, and STPH. LASSO, least absolute shrinkage and selection operator; TCGA, The Cancer Genome Atlas; BC, bladder cancer; NMIBC, non-muscle-invasive bladder cancer; MIBC, muscle-invasive bladder cancer; OS, overall survival; DFS, disease-free survival; STPH, Shanghai Tenth People’s Hospital. [file Image_1.jpeg]

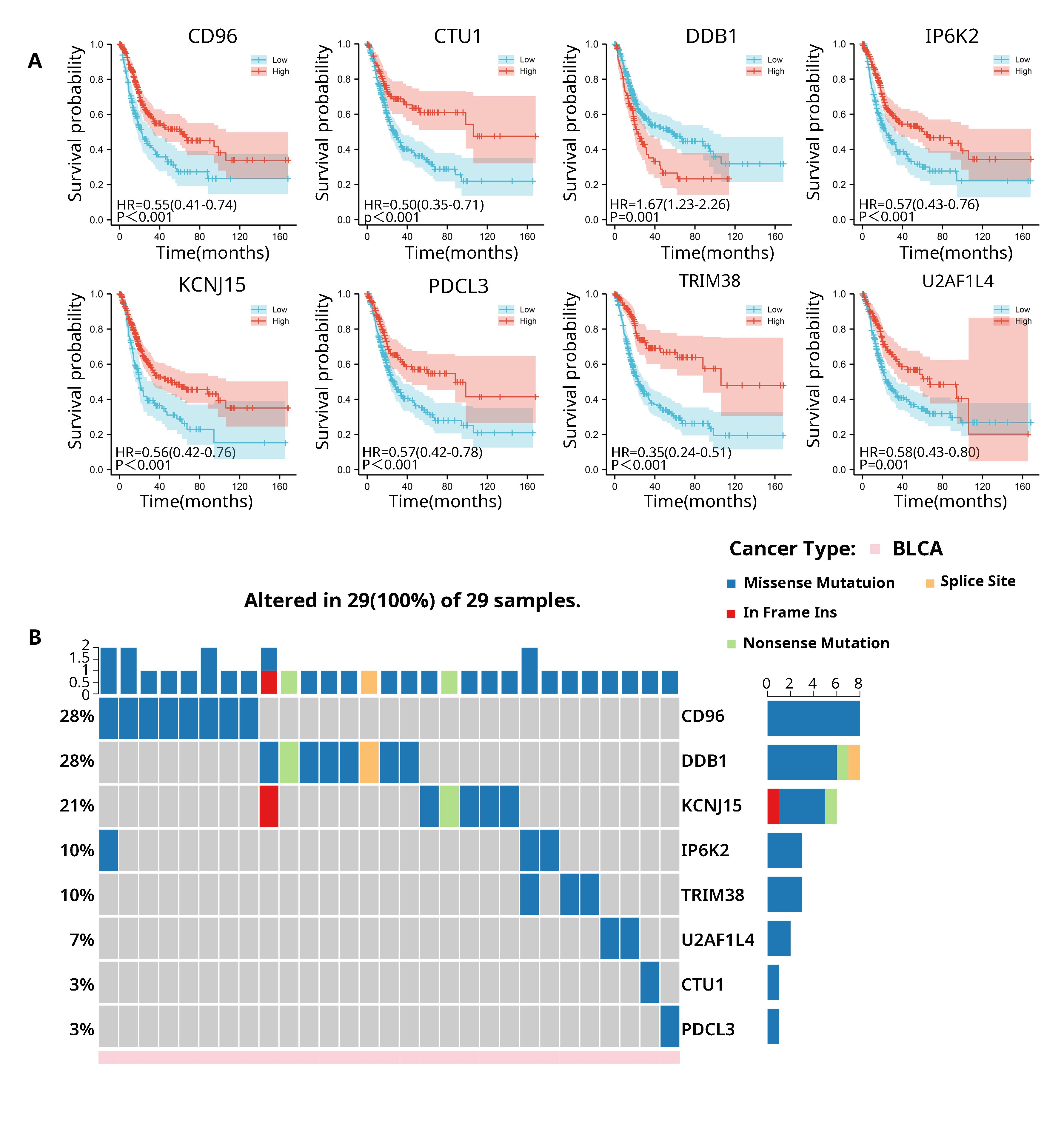

Supplement: Supplementary Figure 2 — The prognostic ability and single nucleotide variation of eight genes using TCGA datasets. (A) Kaplan-Meier curves for overall survival of eight genes individually. (B) Single nucleotide variation distribution among eight genes. BC, bladder cancer. [file Image_2.jpeg]

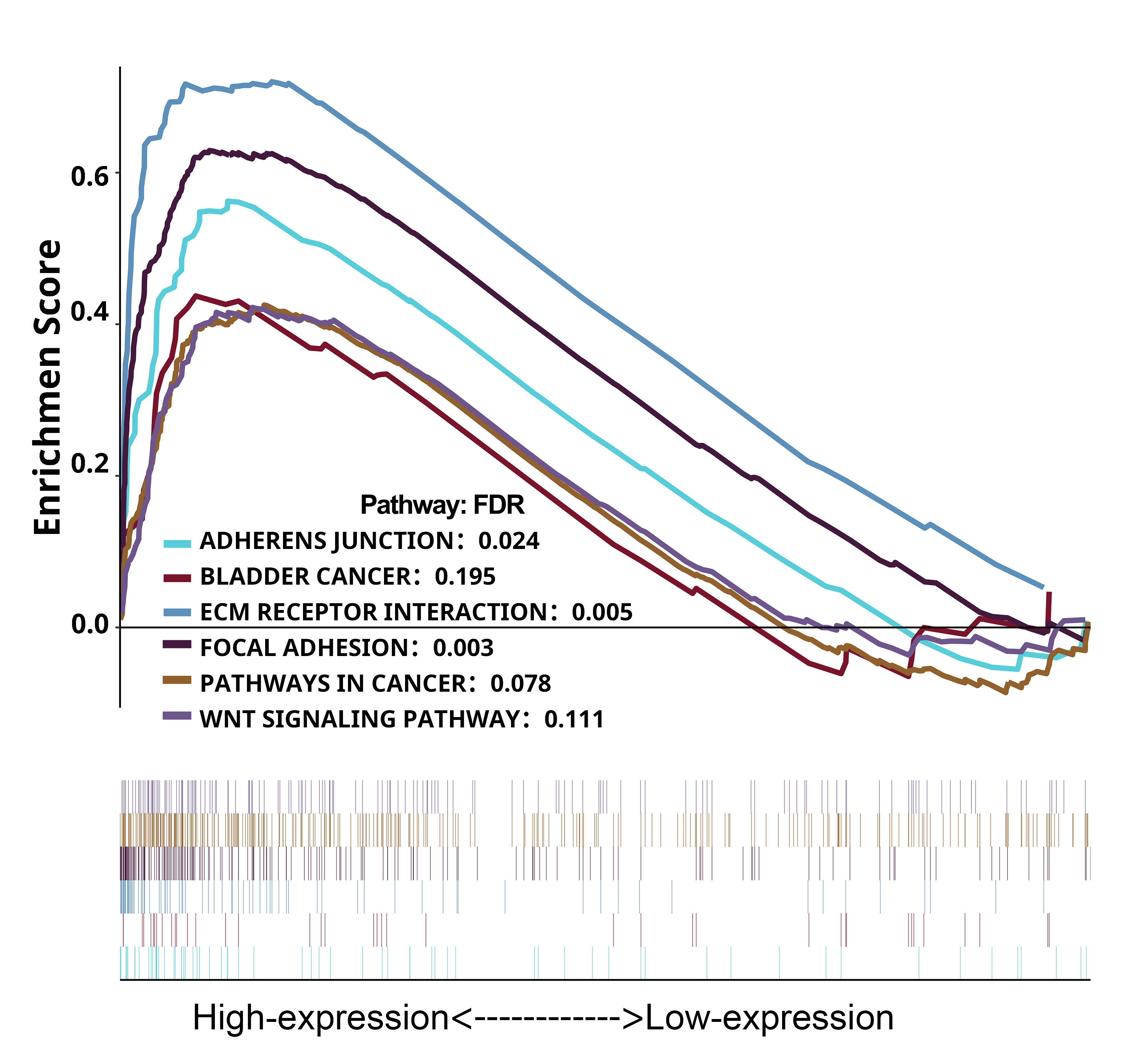

Supplement: Supplementary Figure 3 — Gene Set Enrichment Analysis between patients in high- and low-risk groups. BC, bladder cancer. [file Image_3.jpeg]
