# Supplementary material for: Construction and Validation of a Novel Eight-Gene Risk Signature to Predict the Progression and Prognosis of Bladder Cancer
Source: Front Oncol. 2021 Jun 29;11:632459. doi: 10.3389/fonc.2021.632459 (PMC8276675; doi:10.3389/fonc.2021.632459)
Supplement: Supplementary file 4 [file Table_1.docx]

Supplement Table 1: Characteristic of patients with bladder cancer in TCGA and STPH.

| Characteristic, n (%) | TCGA (n=403) | STPH (n=49) |
| --- | --- | --- |
| Age (years) |  |  |
| ≥65 | 254 (63.0) | 34 (69.4) |
| <65 | 149 (37.0) | 15 (30.6) |
| Sex |  |  |
| Male | 299 (74.2) | 40 (81.6) |
| Female | 104 (25.8) | 9 (18.4) |
| Grade |  |  |
| Low-grade | 21 (5.2) | 7 (14.3) |
| High-grade | 378 (93.8) | 42 (85.7) |
| Unknown | 3 (0.7) | 0 |
| Subtype |  |  |
| NMIBC | 3 (0.7) | 35 (71.4) |
| MIBC | 400 (99.3) | 14 (28.6) |

TCGA: The Cancer Genome Atlas Program; STPH: Shanghai tenth people’s hospital.
