# Supplementary material for: Construction and Validation of a Novel Eight-Gene Risk Signature to Predict the Progression and Prognosis of Bladder Cancer
Source: Front Oncol. 2021 Jun 29;11:632459. doi: 10.3389/fonc.2021.632459 (PMC8276675; doi:10.3389/fonc.2021.632459)
Supplement: Supplementary file 5 [file Table_2.docx]

Supplement Table 2: Primers used for quantitative PCR.

| Promer | Sequence (5'→3') | |
| --- | --- | --- |
| CD96 | F: | CGGAAGGCAAGAAGGAGCACATC |
|  | R: | TTGGGAGAGGTCAGAGGTGGAATG |
| PDCL3 | F: | TCAGAGCAGCCTCAGCAGACC |
|  | R: | TGACGCAGGAGAATCGCTTGAAC |
| IP6K2 | F: | GAGGCAGGAGAATGGCGTGAAC |
|  | R: | GCTCAGGCTGGTCTTGAACTCTTG |
| TRIM38 | F: | CCCACCTCAGCCTCCCAAGTAG |
|  | R: | CCTCTGCTTCACCTTCCACAACC |
| U2AF1L4 | F: | CATCTTTGCCAGTCCCTTCCATCC |
|  | R: | CGGTACAGGTTGAGCAGCACTATG |
| DDB1 | F: | CAGTATGACGATGGCAGCGGTATG |
|  | R: | GGAAGGAGGACAACTGGCAACAC |
| KCNJ15 | F: | ACCTCCTCCCTGACCACAACTATG |
|  | R: | TGTAAGCAGCCACTTCGCAATCC |
| CTU1 | F: | AGGCATCTGTGGCTGAGGAAGG |
|  | R: | GTGACGGCGGCTGTGAATGG |
| †F,forward; R,reverse | | |
